# Novel Liver Stiffness-Based Nomogram for Predicting Hepatocellular Carcinoma Risk in Patients with Chronic Hepatitis B Virus Infection Initiating Antiviral Therapy

**DOI:** 10.3390/cancers13235892

**Published:** 2021-11-23

**Authors:** Jae Seung Lee, Hyun Woong Lee, Tae Seop Lim, Hye Jung Shin, Hye Won Lee, Seung Up Kim, Jun Yong Park, Do Young Kim, Sang Hoon Ahn, Beom Kyung Kim

**Affiliations:** 1 Department of Internal Medicine, Yonsei University College of Medicine, Seoul 03722, Korea; sikarue@yuhs.ac (J.S.L.); lhwdoc@yuhs.ac (H.W.L.); tslim21@yuhs.ac (T.S.L.); lorry-lee@yuhs.ac (H.W.L.); ksukorea@yuhs.ac (S.U.K.); drpjy@yuhs.ac (J.Y.P.); dyk1025@yuhs.ac (D.Y.K.); ahnsh@yuhs.ac (S.H.A.); 2 Institute of Gastroenterology, Yonsei University College of Medicine, Seoul 03722, Korea; 3 Yonsei Liver Center, Severance Hospital, Seoul 03722, Korea; 4 Department of Internal Medicine, Gangnam Severance Hospital, Yonsei University College of Medicine, Seoul 06273, Korea; 5 Department of Internal Medicine, Yongin Severance Hospital, Yonsei University College of Medicine, Yongin-si 16995, Gyeonggi-do, Korea; 6 Biostatistics Collaboration Unit, Department of Biomedical Systems Informatics, Yonsei University College of Medicine, Seoul 03722, Korea; hjshin105@yuhs.ac

**Keywords:** hepatitis B virus, antiviral therapy, hepatocellular carcinoma, risk, liver stiffness, nomograms

## Abstract

**Simple Summary:**

We developed a novel risk-scoring model for hepatocellular carcinoma development in treatment-naïve patients with chronic hepatitis B virus infection who are starting antiviral therapy with entecavir or tenofovir. The model reflects age, platelet count, hepatitis B e antigen positivity, serum albumin and total bilirubin levels, cirrhosis development, and liver stiffness values measured by transient elastography. Our new model showed better performance for predicting hepatocellular carcinoma development (Harrell’s *c*-index: 0.799) than the PAGE-B, modified PAGE-B, and modified REACH-B models in Asian patients with chronic hepatitis B receiving potent antiviral therapy.

**Abstract:**

Hepatocellular carcinoma (HCC) risk prediction is important to developing individualized surveillance approaches. We designed a novel HCC prediction model using liver stiffness on transient elastography for patients receiving antiviral therapy against hepatitis B virus (HBV) infection. We recruited 2037 patients receiving entecavir or tenofovir as first-line antivirals and used the Cox regression analysis to determine key variables for model construction. Within 58.1 months (median), HCC developed in 182 (8.9%) patients. Patients with HCC showed a higher prevalence of cirrhosis (90.7% vs. 45.9%) and higher liver stiffness values (median 13.9 vs. 7.2 kPa) than those without. A novel nomogram (score 0–304) was established using age, platelet count, cirrhosis development, and liver stiffness values, which were independently associated with increased HCC risk, along with hepatitis B e antigen positivity and serum albumin and total bilirubin levels. Cumulative HCC probabilities were 0.7%, 5.0%, and 22.7% in the low- (score ≤87), intermediate- (88–222), and high-risk (≥223) groups, respectively. The c-index value was 0.799 (internal validity: 0.805), higher than that of the PAGE-B (0.726), modified PAGE-B (0.756), and modified REACH-B (0.761) models (all *p* < 0.05). Our nomogram showed acceptable performance in predicting HCC in Asian HBV-infected patients receiving potent antiviral therapy.

## 1. Introduction

Globally, approximately 240 million individuals are chronically infected with hepatitis B virus (HBV), which remains a major etiology of hepatocellular carcinoma (HCC) and cirrhosis, especially in endemic areas, including the Republic of Korea [1,2,3]. A high level of serum HBV-DNA is associated with an increased risk of HCC [4,5]; therefore, long-term antiviral therapy (AVT) using potent oral nucleos(t)ide analogs with a high genetic barrier, such as entecavir (ETV) or tenofovir disoproxil fumarate (TDF), was the mainstay of treatment for chronic hepatitis B (CHB) [6,7]. Nevertheless, since AVT does not completely eliminate the risk of HCC development [8], periodic surveillance to detect early-stage HCC is recommended to permit treatment with a curative aim [3,9,10,11]. Accordingly, risk stratification of HCC development among patients with CHB is important for clinicians [12].

Several efforts were made to evaluate HCC development in patients with CHB. Several models, such as the GAG-HCC, CU-HCC, and REACH-B, were designed with sufficiently good prognostic performance [13,14,15]. Although their predictive powers were validated in Asian patients with CHB, they were designed primarily for patients with CHB who were not receiving nucleos(t)ide analogs. However, given that the risk of HCC can be substantially modified by AVT, several models designed for patients receiving nucleos(t)ide analogs, including modified REACH-B (mREACH-B), PAGE-B, and modified PAGE-B (mPAGE-B), were additionally developed with remarkable performance [16,17,18]. Nevertheless, there are still a few unmet needs in the application of these models in real-life clinical practice: Above all, many of these newly suggested models generally include cirrhosis as an essential component. However, the diagnosis of cirrhosis is usually based on routine imaging and/or clinical parameters, and this can be somewhat inaccurate and subject to intra- and interobserver variabilities. To overcome this, the introduction of noninvasive fibrosis tests, such as transient elastography, could further refine the prediction of HCC risk.

In the current era of potent AVT, we aimed to establish a novel prediction model for HCC development optimized for patients with CHB receiving ETV and TDF based on liver stiffness on transient elastography, one of the most reliable fibrosis markers, and validate its role in comparison with that of other prediction models.

## 2. Materials and Methods

### 2.1. Study Design and Participants

Between 2007 and 2018, patients who started AVT with ETV or TDF against chronic HBV infection at Yonsei University Severance Hospital were consecutively screened for eligibility. The inclusion criteria were as follows: (1) age ≥19 years, (2) patients who received ETV or TDF in first-line AVT, (3) reliable liver stiffness values available, (4) no previous history of HCC at enrollment, (5) no previous history of decompensated cirrhosis with Child-Pugh class C at enrollment, and (6) no history of previous organ transplant. The exclusion criteria were as follows: (1) co-infection with other hepatitis viruses, (2) HCC development within 6 months since AVT initiation, (3) death or orthotropic liver transplant within 6 months since AVT initiation, (4) uncontrolled advanced malignancy at enrollment, and (5) other significant medical illnesses.

AVT was initiated in accordance with the practice guidelines of the Korean Association for the Study of the Liver [19] and the reimbursement guidelines of the National Health Insurance Service of the Republic of Korea. Cirrhosis was histologically or clinically diagnosed as follows: (1) platelet count < 150/×10^3^/μL and imaging findings suggestive of cirrhosis, including a blunted, nodular liver edge accompanied by splenomegaly (>12 cm), or (2) clinical signs of portal hypertension, such as gastroesophageal varices [20].

### 2.2. Clinical Evaluation and Follow-Up

During follow-up, patients underwent routine laboratory testing and analyses of the serum levels of HBV-DNA and other viral markers at 3–6-month intervals. They also underwent abdominal ultrasonography and assay of serum alpha-fetoprotein levels at 6-month intervals to screen for HCC [7,21,22,23,24]. Liver stiffness was measured using transient elastography (FibroScan^®^, EchoSens, Paris, France) in a standard way [25]. Only liver stiffness values with at least 10 valid measurements, a success rate of at least 60%, and an interquartile range (IQR) to median ratio of <30% were considered reliable.

The primary outcome was HCC development. HCC was diagnosed histologically, or clinically by dynamic computed tomography and/or magnetic resonance imaging findings (>1 cm nodules with arterial hypervascularity and portal-/delayed-phase washout) [26,27,28,29].

### 2.3. Statistical Analysis

Continuous variables, such as the laboratory test results, are expressed as medians (IQRs) and were compared using Student’s t-test or the Mann–Whitney *U* test depending on their distribution. Categorical variables are expressed as n (%) and were evaluated using the chi-squared test or Fisher’s exact probability test. The cumulative risk of HCC development was calculated using the Kaplan–Meier method. Patients were censored when they ended follow-up, died without developing liver cancer, or developed extrahepatic carcinoma. Potential risk factors for HCC development were screened using univariate Cox regression analyses, and independent associations were assessed using subsequent multivariate Cox regression analyses. Hazard ratios (HR) and 95% confidence intervals (CI) were calculated using Cox regression analysis.

The risk prediction model and nomogram for HCC development probability were constructed on the basis of potential covariates from the multivariate analyses, as well as known risk factors reported in previous studies [12,13,15]. The discriminatory performance of the HCC prediction model was assessed using Harrell’s c-index and time-dependent area under the curve (TDAUC) at 2, 3, and 5 years after AVT initiation. Model performance was represented graphically using calibration plots, which compared model prediction probability with the actual probability of HCC development. In addition, the discriminatory performance of the new model was compared with that of previous models. The prognostic performance of the new HCC prediction model was verified through internal validation using the bootstrap 1000 times resampling method.

All statistical analyses were conducted using SAS software, version 9.2 (SAS Institute, Cary, NC, USA) and R (V.4.0.4, http://cran.r-project.org/, (accessed on 24 August 2021)). Two-sided *p*-values < 0.05 were considered statistically significant.

## 3. Results

### 3.1. Baseline Characteristics and HCC Development

A total of 2037 patients who started their first-line AVT with ETV or TDF was finally included for analysis. Their baseline characteristics are summarized in Table 1. The median age of the subjects was 50 (IQR 41–57) years (1179 [57.9%] men). Cirrhosis and hepatitis B e antigen (HBeAg) positivity were observed in 1016 (49.9%) and 1025 (50.3%) subjects, respectively, at the index date. ETV and TDF were initiated in 917 (45.0%) and 1120 (55.0%) patients, respectively. Transient elastography revealed a median liver stiffness value of 7.6 (IQR 5.4–12.6) kPa.

### 3.2. Comparison between Patients Who Developed HCC and Those Who Did Not

During the median follow-up period of 58.1 (IQR: 36.5–77.4) months, HCC developed in 182 (8.9%) patients (1.91 per 100 patient-years), and the cumulative 2-, 3-, and 5-year incidence rates of HCC were 2.5% (*n* = 48), 4.9% (*n* = 90), and 9.4% (*n* = 138), respectively. The median HCC-free survival duration was 124.5 (IQR: 109.6–125.3) months.

The patients who developed HCC showed significantly older age (median 50 vs. 49 years), higher prevalence of cirrhosis (90.7% vs. 45.9%), higher liver stiffness values (median 13.9 vs. 7.2 kPa), lower platelet counts (median 122.0 vs. 172.5 × 10^3^/μL), lower alanine aminotransferase (ALT) levels (median 47.5 vs. 48.0 IU/L), lower serum albumin levels (median 4.0 vs. 4.2 g/dL), and prolonged prothrombin time (median international normalized ratio: 1.04 vs. 1.00; all *p* < 0.05) than did those who did not (Table S1).

### 3.3. Independent Predictors for HCC Development

Univariate Cox regression analysis revealed that age, presence of cirrhosis, higher liver stiffness values, lower platelet counts, higher ALT levels, higher total bilirubin levels, lower serum albumin levels, and prolonged prothrombin time were significantly associated with the development of HCC (all *p* < 0.001). Subsequent multivariate analysis revealed that age (40 to 50 years [adjusted HR: 4.900, 95% CI: 1.728–13.900], 50 to 60 years (adjusted HR: 5.238, 95% CI: 1.866–14.706), 60 to 70 years (adjusted HR: 6.318, 95% CI: 2.201–18.137), and ≥70 years (adjusted HR: 6.761, 95% CI: 2.015–22.679), in reference to <40 years), presence of cirrhosis (adjusted HR: 4.301, 95% CI: 2.455–7.537), liver stiffness (kPa) (adjusted HR: 1.018, 95% CI: 1.007–1.030), and platelet count levels <134 × 10^3^/μL (adjusted HR: 1.658, 95% CI: 1.197–2.297) were independently associated with an increased risk of HCC development (all *p* < 0.05) (Table 2).

### 3.4. Establishment and Internal Validation of the New Risk Predicting Nomogram for HCC

**Figure 1:**
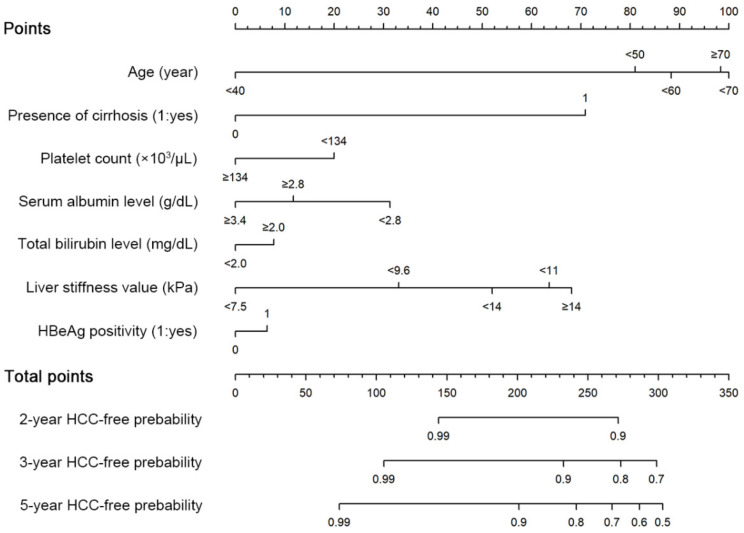
Nomogram for predicting the risk of HCC development. HCC, hepatocellular carcinoma.

**Figure 2:**
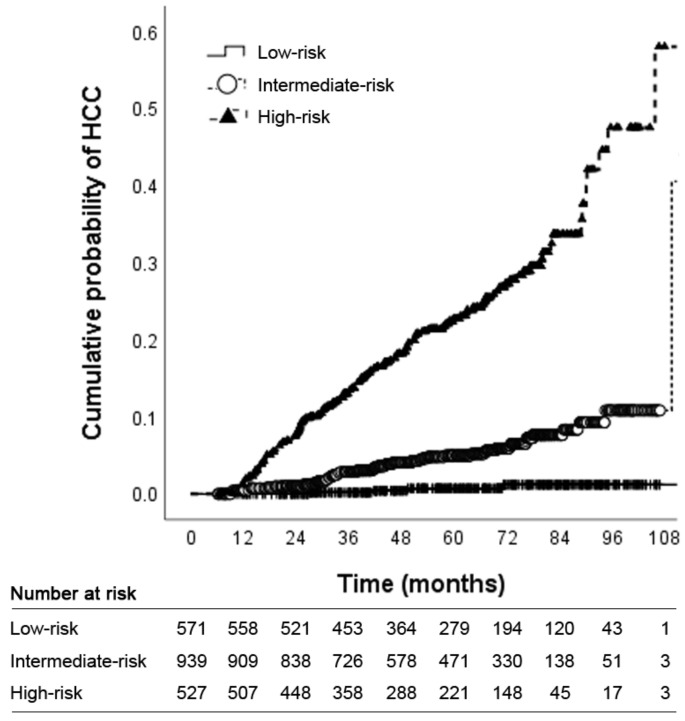
Risk stratification of HCC development according to novel nomogram scores (low-risk, score: ≤87; intermediate-risk, score: 88–222; high-risk: ≥223; *p* < 0.001 by log-rank test). HCC, hepatocellular carcinoma.

Based on the results of multivariate Cox regression analysis, a prediction model and nomogram were developed using the independently associated factors (categorized age, presence of cirrhosis, platelet count, and categorized liver stiffness values [<7.5, 7.5–9.6, 9.6–11.0, 11.0–14.0, and ≥14.0 kPa]), together with categorized serum albumin levels (≥3.4, 2.8–3.4, and <2.8 g/dL), total bilirubin levels (<2.0 and ≥2.0 mg/dL), and HBeAg positivity, which were reported to be closely associated with the HCC risk among subjects with chronic HBV infection [12,13,15]. The model scores ranged from 0 to 304, and when the sum of the scores was more than 74, 201, 242, 267, 287, and 303, the suspected 5-year probabilities of HCC development were 1%, 10%, 20%, 30%, 40%, and 50%, respectively. (Table 3 and Figure 1). The patients were stratified into three groups: low- (score: 0–87, *n* = 571 [28.0%]), intermediate- (score: 88–222, *n* = 939 (46.1%)), and high-risk (score: 223–304, *n* = 527 [25.9%]) groups according to the 25th (87 points) and 75th percentiles (223 points) of the risk score distribution of the nomogram. The 2-, 3-, and 5-year cumulative probabilities of HCC development in the low- (scores: ≤87), intermediate- (88–222), and high-risk (≥223) groups were 0%, 0.2%, and 0.7%; 1.1%, 2.9%, and 5.0%; and 7.5%, 13.3%, and 22.7%, respectively (*p* < 0.001 by the log-rank test; Figure 2).

**Figure 3:**
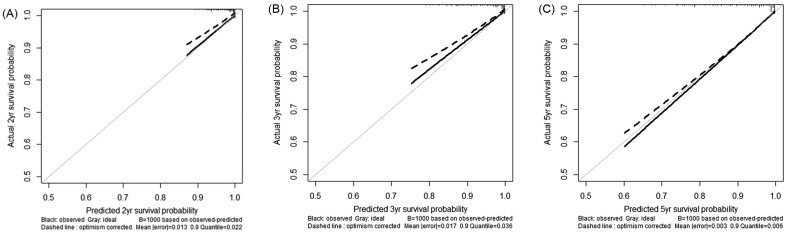
Calibration plot of risk prediction model at 2 (**A**), 3 (**B**), and 5 years (**C**) after initiation of antiviral therapy.

Harrell’s c-index value of the prediction model was 0.799 (95% CI: 0.769–0.829), and the 2-, 3-, and 5-year TDAUCs were 0.802 (95% CI: 0.776–0.827), 0.802 (95% CI: 0.776–0.828), and 0.799 (95% CI 0.773–0.826), respectively (Table 4). Calibration plots for the model for predicting 2-, 3- and 5-year HCC development indicated that the predicted probabilities were very close to observed incidence rates (Figure 3). Subsequent internal validation using bootstrap sampling reproduced similar results: the *c*-index was 0.805 (95% CI: 0.777–0.834), and the 2-, 3-, and 5-year TDAUCs were 0.809 (95% CI: 0.783–0.834), 0.808 (95% CI 0.782–0.834), and 0.805 (95% CI 0.779–0.831), respectively.

### 3.5. Comparison of the New Nomogram and Existing Risk Prediction Models for HCC

The c-index value of the new nomogram for predicting HCC development (0.799) was significantly higher than that of the PAGE-B (0.726, 95% CI: 0.691–0.760, *p* < 0.001), mPAGE-B (0.756, 95% CI: 0.725–0.787, *p* = 0.007), and mREACH-B (0.761, 95% CI: 0.730–0.792, *p* = 0.009) models. The 2-, 3-, and 5-year TDAUCs of the new nomogram (0.802, 0.802, and 0.799) were also significantly higher than those of the PAGE-B (0.712, 0.711, and 0.706), mPAGE-B (0.739, 0.736, and 0.729), and mREACH-B (0.741, 0.738, and 0.738, respectively; all *p* < 0.001) models (Table 4). Furthermore, Harrell’s c-index and 2-, 3-, and 5-year TDAUCs of the “presence of cirrhosis” itself were 0.700 (95% CI: 0.674–0.726) and 0.707 (95% CI: 0.684–0.729), 0.707 (95% CI: 0.683- 0.730), and 0.704 (95% CI 0.680–0.728), respectively, which were significantly lower than those of our nomogram (all *p* < 0.001).

### 3.6. External Validation of the New Nomogram for HCC in an Independent Cohort

For external validation, we recruited 901 patients from Gangnam Severance Hospital and Yongin Severance Hospital using the same criteria. During follow-up (median: 51.8 [IQR 34.4–73.1] months), HCC developed in 56 (6.2%): 10 patients without cirrhosis and 46 patients with cirrhosis in this cohort. The c-index value and 2-, 3-, and 5-year TDAUC of the nomogram were also acceptable: 0.785 (95% CI: 0.729– 0.840) and 0.782 (95% CI: 0.722–0.841), 0.777 (95% CI: 0.719–0.835), and 0.771 (95% CI: 0.714–0.827), respectively, in overall patients. In subgroup analysis, Harrell’s C-index, 2-, 3-, and 5-year TDAUC values were 0.749 (95% CI: 0.692–0.806), 0.649 (95% CI: 0.522–0.775), 0.651 (95% CI: 0.524–0.779), and 0.651 (95% CI: 0.525–0.776), respectively, in patients without cirrhosis and 0.668 (95% CI: 0.585–0.750), 0.653 (95% CI: 0.571–0.736), 0.651 (95% CI: 0.570–0.732), and 0.653 (95% CI: 0.573–0.733), respectively, in patients with cirrhosis (*n* = 324, 36.0%).

## 4. Discussion

Several risk-scoring systems were proposed for HBV-related HCC, most of which generally exhibit high negative predictive values to exclude HCC development in the next 3–10 years [12]. Based on accumulated evidence that potent nucleos(t)ide analogs, such as ETV and tenofovir, can considerably decrease the risk of HCC development, recommended indications of AVT suggested by practice guidelines are gradually widening [6]. Therefore, in the current era of potent AVT, the constant application of existing HCC prediction models without proper interpretation of viral and host factors, including intrahepatic fibrotic burden, would not provide information applicable to actual clinical practice. Herein, we established a novel risk-scoring model and nomogram for HCC development using clinical variables widely available in real-world practice, and then compared the performance of our model with that of previous models.

Our study has several strengths. Firstly, our nomogram showed consistently superior c-index and TDAUC values for predicting HCC development during the long-term follow-up period, compared with that of other conventional risk prediction models, including the PAGE-B, mPAGE-B, and mREACH-B models, which showed high performance in previous literature [12,16,30]. Considering that cirrhosis itself alone can have potent discrimination power in predicting HCC risk with moderate prognostic performances, the performance of the nomogram in a subgroup with cirrhosis was slightly suboptimal. Nevertheless, owing to the adoption of other components, including liver stiffness, the overall predictive power could be significantly enhanced, showing their mutually complementary relationship. In addition, calibration plots of our nomogram indicated that the model provided unbiased result estimates. Moreover, the high performance of our nomogram was maintained in external validation. The PAGE-B and mPAGE-B models employed platelet counts and serum albumin levels to reflect the fibrotic burden of patients with chronic HBV infection on treatment with AVT. In contrast, our nomogram included key variables that more comprehensively cover an individual patient’s fibrotic burden (i.e., presence of cirrhosis, liver stiffness value, and platelet count), and the incorporation of serum albumin and bilirubin levels allow for the assessment of hepatic functional reserve related with the hepatic function of protein synthesis and detoxification. Such differences from the previous models may underlie the outstanding performance of our nomogram. Secondly, the large sample of >2000 patients who underwent baseline transient elastography with a long-term follow-up, as well as the sufficient number of HCC cases (*n* = 182, 8.9%) during the long-term follow-up period of approximately 60 months, allowed for statistical reliability and adequate power [18,31]. The higher HCC development during a follow-up period of 58.1 months may be derived from the higher prevalence of cirrhosis, compared to that shown in the study based upon a nationwide cohort (49.9% vs. 34.4%) [32]. Finally, in a similar context, the homogeneous distribution of our study population in terms of ethnicity, antiviral regimens (ETV or TDF), and HBV genotype C2 (>98%) might be another advantage. Therefore, our study results can be applied to real-world clinical settings, despite the retrospective study design.

The 5-year cumulative incidence of HCC was <1% in the low-risk group (score: ≤87). Generally, the currently recommended bi-annual surveillance strategy is cost-effective when the annual incidence rate of HCC ranges ≥0.2% in patients with chronic HBV infection and ≥1.5% in those with cirrhosis [33,34]. Therefore, in the low-risk group (*n* = 571, 28.0%), HCC surveillance can be mitigated safely, until the value calculated by our nomogram reaches more than 87. Accordingly, repeated assessment of HCC risk at every visit is required in routine practice. Contrary, because the intermediate- and high-risk groups (46.1% and 25.9%, respectively) had significantly higher annual incidence rates of HCC (1.0% and 4.5%, respectively), a more sensitive imaging study of detecting HCC rather than abdominal ultrasonography, for example, magnetic resonance imaging with or without contrast, is required. Further prospective studies on personalized surveillance strategies according to individual risk and cost-effectiveness are required.

The type of AVT does not seem to influence the risk of HCC development. Our study included similar proportions of patients who first started AVT with ETV (45.0%) and TDF (55.0%), and the type of AVT was not associated with HCC development in univariate analysis. There is controversy on whether TDF is more advantageous than ETV in reducing HCC risk [35]; however, recently published studies support that the cumulative HCC risk does not change depending on the type of AVT [36,37]. Moreover, a recent study revealed that ETV also showed a similar risk of HCC development when compared to the newly developed tenofovir alafenamide (TAF), a substitute for TDF based on its proven efficacy and safety. [38,39]

Unsolved issues remain in our study. Firstly, the findings of this study were potentially subject to selection bias. Considering that our institute is the second largest tertiary hospital with approximately 2500 beds in the Republic of Korea, our study population was more likely to cover patients with advanced liver disease and a high prevalence of cirrhosis in comparison with the nationwide cohort [32]. However, we attempted to overcome the selection bias by recruiting a homogeneous study population with a statistically reliable large sample size and event number, employing a longer follow-up duration, and comprehensively using various parameters easily available in routine clinical practice with optimized statistical approaches. However, our hypothesis should be validated through additional studies. Second, the enrollment of patients treated with TAF, a similarly potent and safer agent in terms of renal function and bone mineral density than TDF, would have provided more generalizable results representative of global HBV-infected populations. However, since TAF has only been officially reimbursed in the Republic of Korea since November 2017, the follow-up data of patients with chronic HBV infection who were firstly treated with TAF were not adequate to observe a sufficient number of liver-related events. Nevertheless, since the difference in the preventive effect on HCC development among the three antiviral regimens is still a controversial issue, further studies are required. Finally, the evaluation of new biomarkers for chronic HBV infection (e.g., quantitative HBV surface antigen, serum HBV-RNA, hepatitis B core-related antigen, or specific HBV mutants) was limited due to the retrospective nature of our study [40,41,42]. Notwithstanding, previous studies have shown associations with HCC development for high quantitative surface antigen or high core-related antigen levels [40,43]. Although assays were developed to make serum analysis of these markers possible, sufficient data were not yet established due to the relatively recent introduction thereof.

## 5. Conclusions

This study developed a novel nomogram to predict HCC using baseline information readily available among treatment-naïve patients with chronic HBV infection who started their first-line AVT with potent nucleos(t)ide analogs. The nomogram consistently showed better prognostic performance over that of conventional models. Further studies are required to validate our results among independent cohorts, including Western populations.

## Figures and Tables

**Table 1:** Baseline characteristics of study population.

Variable	Total (*n* = 2037)
Age (years)	50 (41–57)
<40	468 (23.0)
40–50	531 (26.1)
50–60	681 (33.4)
60–70	289 (14.2)
≥70	68 (3.3)
Male sex	1179 (57.9)
Presence of cirrhosis	1016 (49.9)
HBeAg positivity	1025 (50.3)
TDF use (vs. ETV)	1120 (55.0)
Liver stiffness value ^†^ (kPa)	7.6 (5.4–12.6)
Laboratory test results	
Platelet count (×10^3^/μL)	168.0 (123.0–213.0)
AST level (IU/L)	44.0 (28.0–82.5)
ALT level (IU/L)	48.0 (26.0–111.0)
Total bilirubin level (mg/dL)	0.8 (0.6–1.1)
Serum albumin level (g/dL)	4.2 (3.9–4.4)
Prothrombin time (INR)	1.00 (0.95–1.07)
Alpha-fetoprotein level (ng/mL)	4.01 (2.60–7.79)

Values are expressed as a *n* (%) or median (interquartile range). ^†^ Measured using transient elastography (FibroScan^®^, EchoSens, Paris, France. TDF, tenofovir disoproxil fumarate; ETV, entecavir; HBeAg, hepatitis B e antigen; AST, aspartate aminotransferase; ALT, alanine aminotransferase; INR, international normalized ratio.

**Table 2:** Multivariate Cox regression analysis for development of hepatocellular carcinoma.

Variable	Univariate *p* Value	Multivariate Analysis	
		*p* Value	Hazard Ratio (95% CI)
Age (year)			
<40	-	-	Reference
40–50	<0.001	0.003	4.900 (1.728, 13.900)
50–60	<0.001	0.002	5.238 (1.866, 14.706)
60–70	<0.001	0.001	6.318 (2.201, 18.137)
≥70	<0.001	0.002	6.761 (2.015, 22.679)
Presence of cirrhosis	<0.001	<0.001	4.301 (2.455, 7.537)
Liver stiffness value ^†^ (kPa)	<0.001	0.002	1.018 (1.007, 1.030)
Platelet count <134×10^3^/μL	<0.001	0.002	1.658 (1.197, 2.297)
ALT level ≥80 IU/L	<0.001	0.604	1.106 (0.756, 1.617)
Total bilirubin level ≥2.0 mg/dL	<0.001	0.980	0.993 (0.582, 1.695)
Serum albumin level <3.4 g/dL	<0.001	0.250	1.307 (0.828, 2.061)
Prothrombin time (INR) ≥1.5	<0.001	0.601	1.187 (0.624, 2.259)

^†^ Continuous variable, measured using transient elastography (FibroScan^®^, EchoSens, Paris, France). CI, confidence interval; ALT, alanine aminotransferase; INR, international normalized ratio.

**Table 3:** Novel nomogram for risk prediction of hepatocellular carcinoma development.

Covariates	Points
Age (reference: <40 years)	0
40–50 years	81
50–60 years	88
60–70 years	100
≥70 years	98
Cirrhosis (reference: no)	0
Yes	71
Platelet count (reference: ≥134 × 10^3^/μL)	0
<134 × 10^3^/μL	20
Serum albumin (reference: ≥3.4 g/dL)	0
2.8–3.4 g/dL	12
<2.8 g/dL	31
Total bilirubin (reference: <2.0 mg/dL)	0
≥2.0 mg/dL	8
HBeAg (reference: negative)	0
Positive	6
Liver stiffness value ^†^ (reference: <7.5 kPa)	0
7.5–9.6 kPa	33
9.6–11.0 kPa	64
11.0–14.0 kPa	52
≥14.0 kPa	68

^†^ Measured using transient elastography (FibroScan^®^, EchoSens, Paris, France). HBeAg, hepatitis B e antigen.

**Table 4:** Comparison of predictive performance for HCC risk among new nomogram, PAGE-B, modified PAGE-B, and modified REACH-B models in derivation cohort.

	Nomogram (95% CI)	PAGE-B (1) (95% CI)	Modified PAGE-B (2) (95% CI)	Modified REACH-B (3) (95% CI)	*p* Value (Nomogram vs.)		
					(1)	(2)	(3)
Harrell’s c-index	0.799 (0.770, 0.827)	0.726 (0.691, 0.760)	0.756 (0.725, 0.787)	0.761 (0.730, 0.792)	<0.001	0.007	0.009
TDAUC at 2 year	0.802 (0.776, 0.827)	0.712 (0.680, 0.744)	0.739 (0.712, 0.767)	0.741 (0.714, 0.769)	<0.001	<0.001	<0.001
TDAUC at 3 year	0.802 (0.776, 0.828)	0.711 (0.678, 0.744)	0.736 (0.708, 0.763)	0.738 (0.711, 0.766)	<0.001	<0.001	<0.001
TDAUC at 5 year	0.799 (0.773, 0.826)	0.706 (0.674, 0.738)	0.729 (0.703, 0.756)	0.738 (0.711, 0.765)	<0.001	<0.001	<0.001

HCC, hepatocellular carcinoma; CI, confidence interval; TDAUC, time-dependent area under receiver operational characteristics curve.

## Data Availability

The data presented in this study are available on request from the corresponding author. The data are not publicly available due to patient privacy concerns.

## Supplementary Materials

The following are available online at https://www.mdpi.com/article/10.3390/cancers13235892/s1, Table S1: Comparison of the baseline characteristics between the patients who developed HCC and those who did not.
