# Supplementary material for: Novel Liver Stiffness-Based Nomogram for Predicting Hepatocellular Carcinoma Risk in Patients with Chronic Hepatitis B Virus Infection Initiating Antiviral Therapy
Source: Cancers (Basel). 2021 Nov 23;13(23):5892. doi: 10.3390/cancers13235892 (PMC8656676; doi:10.3390/cancers13235892)
Supplement: Supplementary file 1 [file cancers-13-05892-s001.zip › cancers-1394915-supplementary.pdf]

**Table S1.** Comparison of the baseline characteristics between the patients who developed HCC and those who did not.

| Variable | Non-HCC (*n*=1855, 91.1%) | HCC (*n*=182, 8.9%) | *p* Value |
|---|---|---|---|
| Age (years) | 49 (39–56) | 55 (49–60) | <0.001 |
| <40 | 464 (25.0) | 4 (2.2) | |
| 40–50 | 485 (26.1) | 46 (25.3) | |
| 50–60 | 600 (32.3) | 81 (44.5) | <0.001 |
| 60–70 | 247 (13.3) | 42 (23.1) | |
| ≥70 | 59 (3.2) | 9 (4.9) | |
| Male sex | 1,063 (57.3) | 116 (63.7) | 0.940 |
| Presence of cirrhosis | 851 (45.9) | 165 (90.7) | <0.001 |
| HBeAg positivity | 936 (50.5) | 89 (48.9) | 0.683 |
| TDF use (vs. ETV) | 1,030 (55.5) | 90 (49.5) | 0.116 |
| Liver stiffness value† (kPa) | 7.2 (5.2–11.8) | 13.9 (9.8–21.9) | <0.001 |
| Laboratory test results | | | |
| Platelet count (×10$^3$/μL) | 172.5 (128.0–217.0) | 122.0 (94.0–161.8) | <0.001 |
| AST level (IU/L) | 43.0 (27.0–83.0) | 50.0 (37.0–80.0) | 0.582 |
| ALT level (IU/L) | 48.0 (25.0–115.0) | 47.5 (30.0–84.3) | <0.001 |
| Total bilirubin level (mg/dL) | 0.8 (0.6–1.1) | 0.9 (0.7–1.4) | 0.061 |
| Serum albumin level (g/dL) | 4.2 (3.9–4.4) | 4.0 (3.5–4.2) | 0.019 |
| Prothrombin time level (INR) | 1.00 (0.95–1.07) | 1.04 (0.99–1.14) | <0.001 |
| Alpha-fetoprotein level (ng/mL) | 3.81 (2.51–7.24) | 6.89 (4.41–13.66) | 0.324 |

Values are expressed as *n* (%) or median (interquartile range). †Measured using transient elastography (FibroScan®, EchoSens, Paris, France). HCC, hepatocellular carcinoma; TDF, tenofovir disoproxyl fumarate; ETV, entecavir; HBeAg (+), positive for hepatitis B e antigen; AST, aspartate aminotransferase; ALT, alanine aminotransferase; INR, international normalized ratio; HBV, hepatitis B virus.
